# Knowledge and Attitude Toward Vital Pulp Therapy Among Dental Practitioners in Jordan: A Questionnaire-Based Survey

**DOI:** 10.1155/ijod/3969832

**Published:** 2025-09-08

**Authors:** Rawan Abu Zaghlan, Nessrin Taha, Areej Alqadi, Afnan AlHafnawi, Ahmad Sabri, Rawan Alnahar

**Affiliations:** ^1^Department of Restorative Dentistry, School of Dentistry, The University of Jordan, Amman 11942, Jordan; ^2^Department of Conservative Dentistry, Jordan University of Science and Technology, Irbid, Jordan; ^3^Department of Preventive Dentistry, Jordan University of Science and Technology, Irbid, Jordan

**Keywords:** deep caries, permanent teeth, vital pulp therapy

## Abstract

**Aim:** To explore the current state of awareness and acceptance of vital pulp therapy (VPT) in permanent teeth among dental students and dental practitioners in Jordan.

**Materials and Methods:** An online questionnaire was sent to undergraduate (UG) students, intern dentists, general dental practitioners (GDPs), paediatric dentistry postgraduates (PGs) (Paed. PGs) and endodontics PGs (Endo. PGs) to collect data on their source of information and references about VPT. The survey encompassed assessments of participants' understanding of indications, contraindications, technical steps, materials, complications and factors influencing the success of VPT procedures. Descriptive analysis was performed, and chi-square tests were used to assess correlations (*p* ≤ 0.05).

**Results:** A total of 402 responses were analysed. Of these, 11.4% had never practiced VPT, while the remaining respondents reported practicing it with varying frequencies, ranging from 27.4% on a weekly basis to 23.4% rarely. PGs and GDPs practiced VPT more than other categories (*p* < 0.001), position statements were less relied upon for information. Participants were familiar with indirect pulp capping (IPC) and direct pulp capping (DPC) (91.8% and 87.1%, respectively) more than full (78.9%) and partial pulpotomy (70.4%). Irreversible pulpitis (39.1%) and extensive restorative needs (36.8%) were commonly perceived as contraindications for VPT. For reversible pulpitis, stepwise selective caries removal with IPC was the preferred treatment (30.6%), while root canal treatment (RCT) was preferred for irreversible pulpitis (47.5%). Calcium hydroxide (51.7%) was the preferred material for IPC among UGs and GDPs, while calcium silicate-based materials (34.3%) were favoured by PGs. Mineral trioxide aggregate (MTA) was the material of choice for DPC and pulpotomy (71.6%) across all groups. Sodium hypochlorite (NaOCl) (58.7%) was the preferred material for achieving haemostasis. Most participants (80.1%) were aware of clinical and radiographic outcome assessment and the importance of aseptic technique. Lack of knowledge was the main barrier to VPT, and over 96% expressed interest in further training.

**Conclusions:** While there is a generally positive attitude toward VPT among dental professionals in Jordan, gaps exist in indications, clinical procedures and complications, particularly among UGs and GDPs. This highlights the need for enhanced curricula and continuing education programmes to bridge these gaps and enhance confidence in performing VPT.

**Clinical Relevance:** Improving practitioners' knowledge and confidence in performing VPT could popularize it as a minimally invasive, cost-effective and less technically challenging alternative to RCT in appropriate clinical scenarios. This would ultimately benefit patient outcomes and long-term oral health.

## 1. Introduction

Preservation of dental pulp vitality is crucial in ensuring the long-term health and functionality of permanent teeth [1, 2]. Vital pulp therapy (VPT), encompassing direct pulp capping (DPC), indirect pulp capping (IPC) and partial and complete pulpotomy, has emerged as a contemporary approach to treat and manage inflamed pulps [3]. Previously, the primary emphasis of VPT focused on preserving the radicular pulp in immature teeth to facilitate the completion of root formation, a process known as apexogenesis. Recently, the scope of VPT has expanded significantly. Practitioners now have a broader range of treatment options available to preserve all or part of the pulp, even in cases of mature teeth diagnosed with irreversible pulpitis, which were once believed to require root canal treatment (RCT) as the sole treatment option, since this diagnosis may not accurately represent the true histological status of the pulp [4–6].

A histological study showed that in teeth with clinical symptoms indicative of irreversible pulpitis, inflammation and infection are present in a confined area near the pulp exposure [7]. This indicates that the pulp could be saved by removal of the stimulus and the infected tissues via partial or full pulpotomy [5, 8].

The American Association of Endodontists (AAEs) and the European Society of Endodontology (ESE) have each published position statements on VPT and the management of deep carious lesions [3, 9]. These documents offer evidence-based guidance, including clear definitions, clinical indications and procedural recommendations for practitioners. These documents should be provided for students and made available for practitioners as well.

The dental landscape in Jordan has experienced dynamic changes in recent years, with advancements in education, technology and treatment modalities [10]. As the field continues to evolve, it is essential to understand the perspectives of dental professionals at various career stages. Such insights can help tailor educational strategies, refine clinical practices and enhance the overall quality of patient care [11].

While a few studies have evaluated the knowledge of dental students and practitioners regarding VPT in other countries [12–14], no research has been conducted to assess the knowledge and attitudes of dental students and practitioners in Jordan. This study aimed to investigate the current state of knowledge and attitude toward VPT among dental professionals at different stages of their careers within the Jordanian dental community.

By exploring the existing landscape, we seek to identify potential gaps in understanding, uncover prevalent beliefs and practices and highlight areas that warrant further attention and education within the dental community. Additionally, insights gained from this investigation can pave the way for targeted interventions aimed at fostering a more comprehensive and nuanced approach to VPT, ultimately contributing to improved patient outcomes and the overall advancement of dental healthcare and education in Jordan.

## 2. Materials and Methods

This was a cross-sectional questionnaire-based study approved by the Institutional Review Board/Deanship of Scientific Research at the University of Jordan (52-2023). All participants provided informed consent to participate voluntarily in the questionnaire before answering the questions.

This study was conducted in accordance with the STrengthening the Reporting of OBservational studies in Epidemiology (STROBE) guidelines [15, 16] and utilised the STROBE checklist for cross-sectional studies to guide the study's design and reporting of results.

A structured questionnaire was developed based on current literature and expert opinion. To ensure clarity and readability, the draft questionnaire was reviewed by two endodontists, a paediatric dentist and three dental interns. Revisions were made based on their feedback to refine the wording and improve comprehension.

The final version of the questionnaire was created in English. A 22-item multiple-choice questionnaire was devised and assembled utilising an online survey platform (Google Forms; https://www.google.com/forms/about/). The initial section comprised six inquiries concerning participants' demographic details, including gender, age, education level, country of primary degree, years of professional experience and work sector. The subsequent section included 16 questions probing participants' background knowledge of VPT, sources of information, familiarity with VPT techniques, application frequency, understanding of indications and contraindications, preferences for pulp capping and haemostasis materials, factors influencing treatment success, awareness of potential complications, criteria for determining treatment outcome, reasons for refraining from offering VPT, preferred treatments for various clinical scenarios and interest in attending courses related to VPT techniques.

The questionnaire was piloted on a 5% sample of the participants to identify potential difficulties during data collection and to assess its readability and clarity.

Participation in the study was voluntary, with confidentiality and anonymity assured to respondents. A survey link was created and distributed to dental students and interns at two major dental institutions in Jordan, as well as to general dental practitioners (GDPs) and postgraduates (PGs) specialising in paediatric dentistry and endodontics across Jordan through social media invitations, including WhatsApp and Facebook groups. With an estimated population of 10,000 dentists in Jordan, a sample size of 370 responses would be sufficient to achieve 95% confidence and a margin of error of 5%, ensuring adequate statistical power.

Descriptive data analysis was carried out. The chi-square test (at the participant's level and probability values ≤ 0.05) was used to assess correlations between demographic characteristics and participants' choices (IBM SPSS Statistics for Windows, Version 25.0).

## 3. Results

### 3.1. Demographics

**Figure 1:**
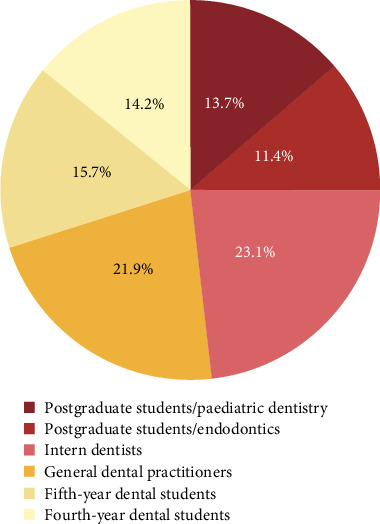
Distribution of survey participants' by educational level.

A total of 402 participants agreed to participate in the survey. The sample consisted of 298 females (74.1%) and 104 males (25.9%) of different educational levels and various work settings. Categories of the participants as students, interns and practitioners are presented in Figure 1.

VPT was practiced by 89% of the respondents but with varying frequencies. Almost a third of participants performed VPT on a weekly basis (27.4%, *N* = 110), followed by 24.1% who performed VPT occasionally (*N* = 97), 23.4% who performed VPT rarely (*N* = 94) and 13.7% who performed VPT monthly (*N* = 55). Only 11.4% of the participants never performed VPT (*N* = 46).

The participants were almost equally distributed between academic and non-academic work settings. In total, 50.5% worked in university hospitals (*N* = 203), 24.1% were in the private sector (*N* = 97), 13.2% in the Ministry of Health (*N* = 53) and 12.2% in the Royal Medical Services (*N* = 49). Almost two-thirds of the participants were aged 20–25 (59.5%, *N* = 239), while 23.4% were aged 26–30. A minority of participants were aged 31–40 (9.7%, *N* = 39), 41–50 (4.7%, *N* = 19) and over 50 (2.7%, *N* = 11).

### 3.2. Familiarity With VPT

**Figure 2:**
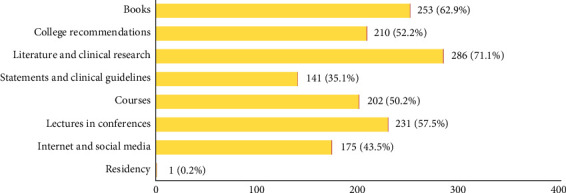
Participants' responses to VPT sources of information.

All participants except one have previously heard about VPT (99.8%, *N* = 401).  Figure 2 illustrates the variable sources of information for participants.

Participants were familiar with all VPT procedures. They were most familiar with IPC and DPC procedures (91.8%, *N* = 369; 87.1%, *N* = 350, respectively) and less familiar with full pulpotomy (78.9%, *N* = 317) and partial pulpotomy (70.4%, *N* = 283). There was no statistically significant difference in familiarity with DPC among age groups. However, there was a statistically significant difference in familiarity with IPC, partial and full pulpotomy procedures. The younger age groups were more familiar with IPC (*p* < 0.001). Participants older than 50 were less familiar with partial pulpotomy compared to younger age groups (*p*=0.013). Moreover, those older than 40 were less familiar with full pulpotomy compared to younger age groups (*p* < 0.001). VPT was more frequently performed by participants aged 26–30, 31−40, 41–50 as opposed to 20–25 and >50 age groups (*p*=0.018).

Likewise, a statistically significant difference in familiarity with IPC, partial and full pulpotomy was found among participants of different educational levels. GDPs and Endo. PGs were less familiar with IPC (*p* < 0.001). Fourth-year UGs, fifth-year UGs, interns and GDPs were less familiar with partial pulpotomy (*p* < 0.001). Also, GDPs and fourth UGs were less familiar with full pulpotomy procedure (*p* < 0.001). VPT was more frequently performed by PGs and GDPs, and most of those who never performed VPT were fourth UGs (*p* < 0.001).

### 3.3. VPT Knowledge and Attitude

• Case selection

Most participants believed that VPT is indicated for traumatic pulp exposure (89.6%, *N* = 360) compared to 68.9% of participants who believed it is indicated for deep/extremely deep caries (*N* = 277) and 67.7% who believed it is indicated for carious pulp exposure (*N* = 272).

While 89.9% and 73.6% of participants believed VPT is contraindicated for necrotic pulp and teeth with periapical radiolucency (*N* = 361 and 296, respectively), 39.1% and 36.8% believed it is contraindicated for teeth with irreversible pulpitis and extensive restorative needs (*N* = 157 and 148, respectively). Only 6.2% of participants considered root maturity to be a contraindication (*N* = 25).

**Figure 3:**
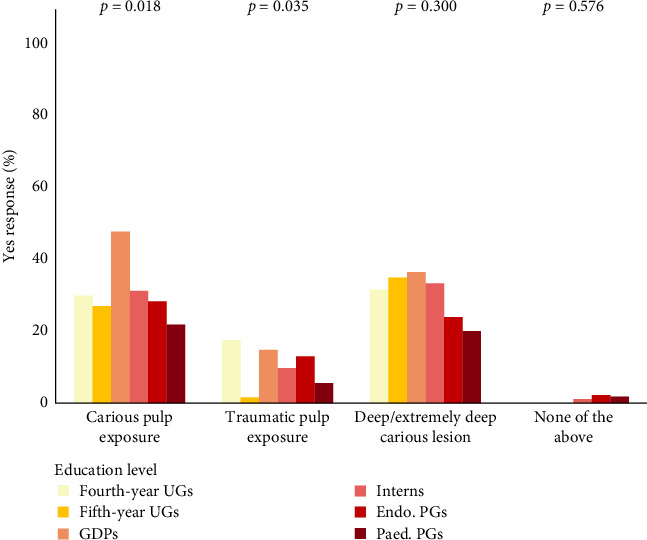
Distribution of participants' responses to VPT indications based on educational level.

**Figure 4:**
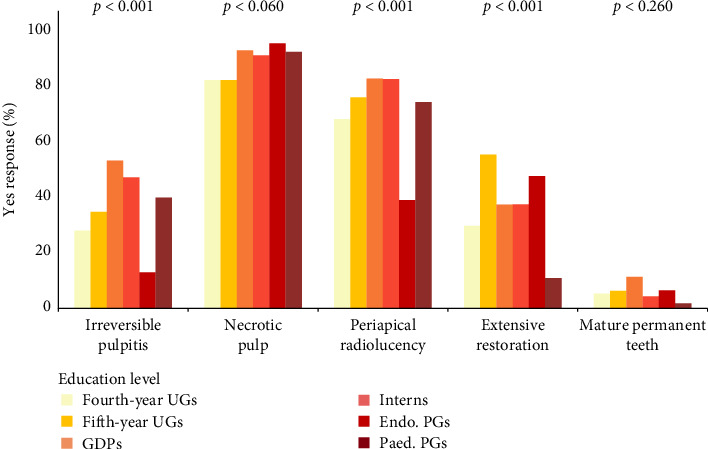
Distribution of participants' responses to VPT contraindication based on educational level.

GDPs were less likely to consider carious pulp exposure as an indication (*p*=0.018) and fourth- year UGs were less likely to consider traumatic pulp exposure as an indication (*p*=0.035). Participants of all educational levels, but Endo. PGs considered periapical radiolucency as a contraindication (*p* < 0.001). Those who considered irreversible pulpitis as a contraindication were mostly GDPs and interns (*p* < 0.001). Extensive restorative needs were a significant contraindication only for fifth-year UGs (*p* < 0.001) (Figures 3 and 4).

When participants were asked about the treatment of choice for extremely deep caries in mature permanent teeth diagnosed with reversible pulpitis, the most preferred treatment option was stepwise selective caries removal with IPC (30.6%, *N* = 123), followed by one-step selective caries removal with IPC (28.6%, *N* = 115). Complete caries removal with DPC, partial or full pulpotomy was less preferred by participants (16.9%, *N* = 68; 12.9%, *N* = 52; 7%, *N* = 28, respectively). The least preferred treatment option was RCT (4%, *N* = 16). While fourth-year UGs, fifth-year UGs, interns and GDPs mostly preferred stepwise selective caries removal with IPC, Paed. PGs mostly preferred one-step selective caries removal with IPC and Endo. PGs mostly preferred complete caries removal with partial pulpotomy (*p* < 0.001).

Conversely, for extremely deep caries in mature permanent teeth diagnosed with irreversible pulpitis, the most opted treatment option was RCT (47.5%, *N* = 191) followed by complete caries removal with full or partial pulpotomy (21.4%, *N* = 86; 17.7%, *N* = 71, respectively). One-step and stepwise selective caries removal with IPC and complete caries removal with DPC were less preferred by participants (2.5%, *N* = 10; 3.7%, *N* = 15; 7.2%, *N* = 29, respectively). While fifth-year UGs, interns, Paed. PGs and GDPs mostly preferred RCT, fourth-year UGs mostly preferred complete caries removal with partial pulpotomy and Endo. PGs mostly preferred complete caries removal with complete pulpotomy (*p* < 0.001).

• Material selection

The material most commonly used for IPC was calcium hydroxide (DyCal) (51.7%, *N* = 208), followed by calcium silicate-based materials, such as mineral trioxide aggregate (MTA)-like materials (34,3%, *N* = 138). Calcium hydroxide was more likely to be used by fourth-year UGs, fifth-year UGs, interns and GDPs, whereas calcium silicate-based materials (MTA-like materials) were more frequently used by Endo. PGs and Paed. PGs (*p* < 0.001). Moreover, calcium hydroxide was the most preferred material for participants aged 20–25 years and 41–50 years, whereas calcium silicate-based materials (MTA-like materials) were mostly preferred by other age groups (*p* < 0.001).

The most preferred material for DPC, partial or full pulpotomy was MTA (71.6%, *N* = 288), among participants of all educational levels and ages (*p* < 0.001, *p*=0.025, respectively).

The material of choice to achieve haemostasis following pulp exposure was NaOCl (58.7%, *N* = 236) followed by a dry cotton pellet (20.1%, *N* = 81) and normal saline (19.7%, *N* = 79). Chlorhexidine was the least preferred material (1.5%, *N* = 6). A tendency to choose NaOCl was evident among participants of all educational levels (*p* < 0.001), and among all age groups except the 41–50 years who mostly preferred a dry cotton pellet (*p* < 0.001).

• VPT outcome assessment and potential prognostic factors

**Figure 5:**
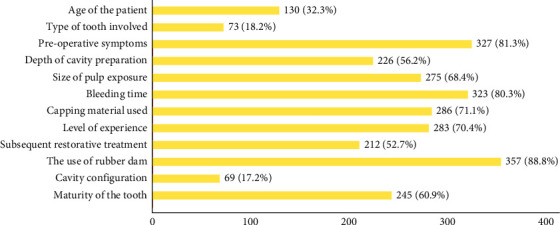
Participants' responses to prognostic factors for VPT.

Factors believed to influence the success of VPT were the use of rubber dam (88.8%, *N* = 357), pre-operative symptoms (81.3%, *N* = 327) and bleeding time (80.3%, *N* = 323) (Figure 5).

The correlations between educational level and determining factors were statistically significant for pre-operative symptoms and the degree of pulp exposure (*p*=0.049 and *p* < 0.001, respectively). Size of pulp exposure was predominantly chosen by fifth-year UGs; however, pre-operative symptoms were least chosen by Endo. PGs.

**Figure 6:**
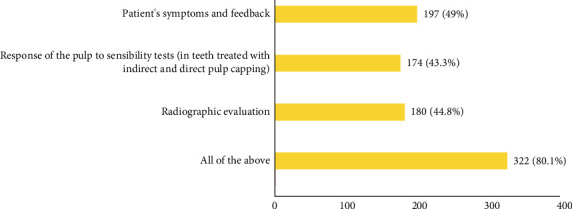
Participants' responses to VPT outcome evaluation criteria.

To evaluate the VPT outcome, most participants would consider patients' symptoms, response to sensibility testing and radiographic evaluation collectively (80.1%, *N* = 322) (Figure 6). The younger age groups (20–25 and 26–30) were more likely to consider all the factors collectively (*p*=0.012) and GDPs were less likely to consider all the factors collectively (*p*=0.042).

For complications of VPT, post-operative pain (79.9%, *N* = 321), pulp necrosis (65.4%, *N* = 263) and tooth discolouration (63.2%, *N* = 254) were frequently chosen. Pulp canal calcification (47.3%, *N* = 190) and root resorption (43.3%, *N* = 174) were less frequently selected.

### 3.4. Barriers to VPT Practice

**Figure 7:**
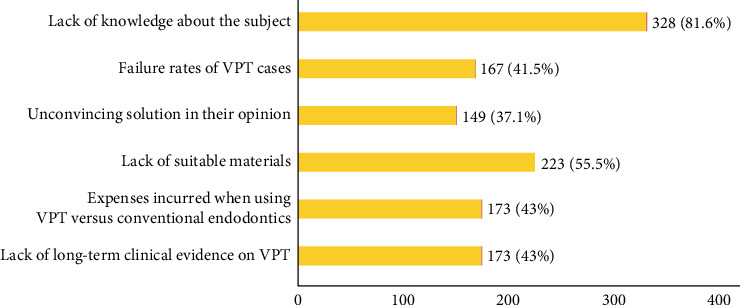
Participants' responses to barriers to VPT practice.

Several factors were considered to refrain dentists from offering VPT as a routine treatment option, with lack of knowledge being the most dominant factor (81.6%, *N* = 328) (Figure 7). Lack of knowledge was perceived as a major barrier for the youngest age groups (20–25 and 26–30) including UGs and PGs (*p*=0.018, *p*=0.014, respectively). Most participants (96.3%, *N* = 387) were interested in attending continuing education courses on clinical techniques and available evidence on the outcome of VPT.

## 4. Discussion

While the results of this survey indicated a 99.8% of participants having prior knowledge of the therapy, there are gaps in knowledge, indications and clinical procedures among the participants. Familiarity with IPC and DPC was highest (91.8% and 87.1%, respectively), while partial and full pulpotomy procedures were less understood (70.4% and 78.9%, respectively). Significant differences were observed across educational levels and clinical experience, with PGs and GDPs demonstrating a more advanced understanding and application of VPT techniques.

### 4.1. Interpretation of Results

The dental profession in Jordan reflects a significant gender shift, with female dentists outnumbering their male counterparts. This trend is mirrored in dental school enrolments, where most dental students are females [17]. The higher proportion of female respondents in the survey reflects this trend, as the sample consisted predominantly of females from diverse educational levels and work settings.

Approximately one-third of participants reported performing VPT on a weekly basis, while fewer than 12% had never carried out the procedure. These figures are considerably higher than those reported in comparable international studies, such as one conducted among Welsh GDPs, where VPT was rarely practiced [18]. This discrepancy could be attributed to active research and curriculum emphasis on minimally invasive endodontic therapies at one of Jordan's major dental schools [19].

The findings of this study also highlight the varying levels of familiarity with VPT procedures across different groups. The strong understanding of IPC and DPC among participants aligns with traditional dental education, as seen in similar studies conducted globally. For instance, in the study from Saudi Arabia, similar trends were observed, where IPC was better understood and more frequently practiced than pulpotomy procedures [14].

The gap in familiarity with the partial and full pulpotomy procedures observed in this study may be linked to the complexity of these treatments, as highlighted by Leong and Yap [20] in their umbrella review. They reported that while pulpotomy procedures, particularly full pulpotomy, have higher success rates, they are often underutilised due to inadequate training [20].

This is consistent with global trends, as shown by findings from other regions around the world. For example, a survey of U.S. dental schools showed that despite the increasing recommendation of VPT, particularly partial and full pulpotomy, its practical teaching remains limited [21]. In China, similar challenges were noted, where the adoption of advanced materials and techniques like partial pulpotomy was lower among less experienced practitioners due to unfamiliarity and perceived operational difficulties [22]. This reflects the situation in Jordan, where less experienced professionals, UGs (fourth and fifth years dental students) and general practitioners are less familiar with advanced VPT techniques, potentially due to gaps in clinical training and exposure.

While calcium hydroxide was the preferred material for IPC, particularly among UGs, calcium silicate materials were favoured for DPC and pulpotomy, with MTA being commonly selected by participants across all educational levels and age groups. This aligns with the growing body of evidence demonstrating that calcium hydroxide is no longer the material of choice for VPT, due to its drawbacks, including high solubility in oral fluids and lack of adhesion to the surrounding tooth structure, in addition to the presence of 'tunnel defects' in the newly formed dentine compromising the integrity of the seal against reinfection [23]. MTA and other calcium silicate-based materials, such as Biodentine and TotalFill, are alternatives for improved clinical outcomes [23].

Only two-thirds of the participants accept VPT for carious pulp exposure, which is alarming in view of available evidence for VPT. Although the AAE and ESE position statements recommend VPT for teeth with clinical signs of irreversible pulpitis, findings show that 39.1% of participants still avoid VPT in such cases, with 36.8% favouring conventional RCT. This confirms the lack of awareness regarding outcome studies and limited familiarity with, or reference to, these guidelines. Successful VPT in mature permanent teeth with carious pulp exposure can be achieved with appropriate case selection and the use of bioactive materials such as MTA, which significantly improves outcomes. This suggests that although VPT is widely recognised, its clinical implementation in Jordan may be hindered by limited understanding and experience with the techniques, an issue echoed in similar studies from other regions [24].

Interestingly, tooth maturity was not widely viewed as a contraindication for performing VPT, with only 6.2% of respondents hesitant to apply the procedure to mature teeth, aligning with current guidelines from professional bodies [3, 9]. In contrast, the presence of periapical radiolucency was generally considered a contraindication for VPT by most participants, except for Endo. PGs.

Traditionally, it was taught that periapical lesions are associated with pulp necrosis; however, it has been shown that periapical inflammatory infiltrates and bone destruction can appear in advance of total pulp necrosis [25]. Several clinical studies reported successful outcomes of VPT in teeth with clinical symptoms of irreversible pulpitis and periapical lesions in both children and adults [4, 26, 27].

A high percentage of participants agreed that post-operative pain is a common complication of VPT in permanent teeth, followed by pulp necrosis and tooth discolouration. Fewer respondents identified pulp canal calcification and root resorption as potential complications. Although pulp canal calcification incidences of 0%–45.0% were reported in previous studies [28].

Sodium hypochlorite was the preferred material for achieving haemostasis among participants across all educational levels and age groups, which aligns with the common use of NaOCl as an antiseptic irrigant in endodontic procedures.

The efficacy of VPT techniques relies heavily on the clinician's knowledge, skills and attitudes [29]. Therefore, by improving awareness and clinical training in VPT amongst dental students and Jordanian dentists, VPT can become more effectively integrated into routine dental practice in Jordan, offering a viable alternative to more invasive treatments, resulting in improved patient outcomes and preservation of dental pulp vitality.

### 4.2. Limitations

This study has several limitations. The response rate could not be determined due to the lack of a prior contact list, potentially introducing sampling bias. The sample was predominantly composed of younger dentists, which may limit the generalizability of the findings. Additionally, using an online questionnaire may cause selection bias, as only individuals with internet access and willingness to participate were included. Nevertheless, this method allowed for a broader range of responses and effectively captured participants' perspectives.

There may be discrepancies between self-reported preferences for VPT and actual clinical practices [17], with possible response bias toward idealised answers. To minimise social desirability bias, data collection was conducted anonymously.

Including UGs, interns, GDPs and PGs provided a wide perspective but also introduced variability in clinical experience and autonomy. Students and interns—who typically work under supervision—may have limited independent decision-making, affecting the consistency of their responses compared to more experienced practitioners.

A key limitation is the absence of multivariate analysis to control for confounders. As this was an exploratory study, the analysis was limited to descriptive and bivariate methods. Future research employing hypothesis-driven designs and multivariable modelling is recommended to better adjust for potential confounders.

### 4.3. Suggestions for Future Research

Future studies should investigate the practical application of VPT techniques through direct observation or clinical audits to provide a more accurate reflection of practices. Further research is also needed to assess the effectiveness of continuing education programmes in bridging the knowledge gap, especially regarding newer materials such as calcium silicate-based cements.

### 4.4. Implications

The findings of this study have significant implications for dental education and clinical practice in Jordan. Educational programmes need to be updated to focus more on management of deep caries via VPT techniques, particularly partial and full pulpotomy. Highlighting its applicability as an alternative technique for management of inflamed pulps in teeth with clinical symptoms of either reversible or irreversible pulpitis.

## 5. Conclusion

The findings of this study highlight a positive attitude toward VPT among dental professionals in Jordan and willingness to learn VPT procedures. However, gaps remain in procedural familiarity and material preferences, especially among UGs and GDPs. Enhanced curricula and continuing education programmes are recommended to bridge knowledge gaps and align practice with evidence-based guidelines. By improving awareness and clinical training, VPT can become more effectively integrated into routine dental practice in Jordan, offering a viable alternative to more invasive treatments, resulting in improved patient outcomes and better preservation of dental pulp vitality.

## Data Availability

The datasets used and/or analysed during this study are available upon request from the corresponding author.

## Nomenclature

IPC: Indirect pulp capping
DPC: Direct pulp capping
VPT: Vital pulp therapy
RCT: Root canal treatment
UGs: Undergraduates
PGs: Postgraduates
Paed. PGs: Paediatric dentistry postgraduates
Endo. PGs: Endodontics postgraduates
GDPs: General dental practitioners
NaOCl: Sodium hypochlorite
MTA: Mineral trioxide aggregate
STROBE: The STrengthening the Reporting of OBservational studies in Epidemiology.
